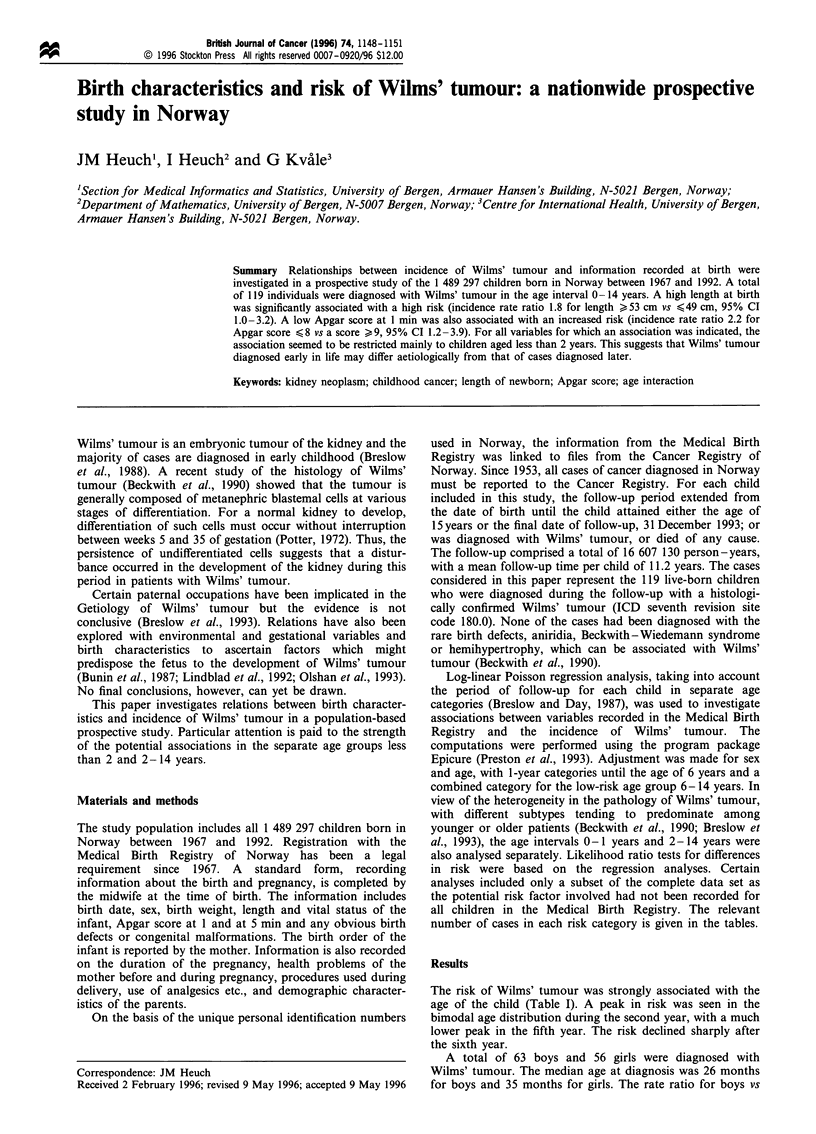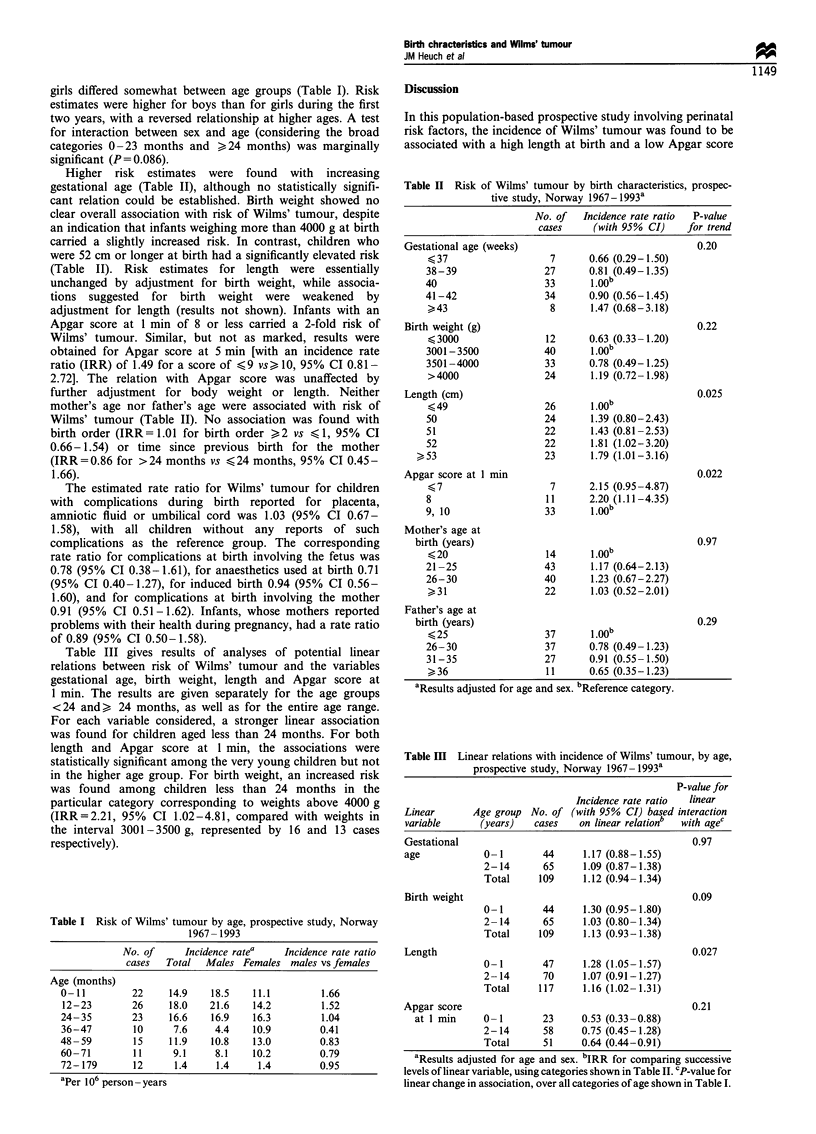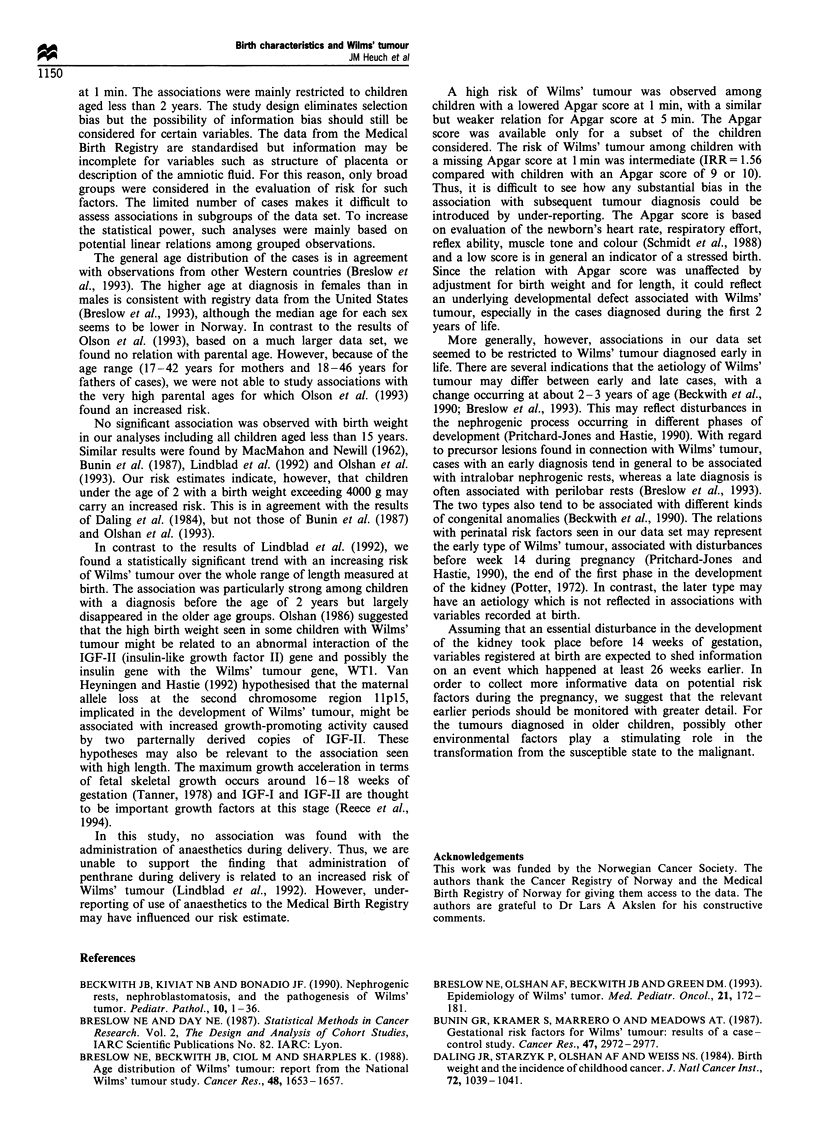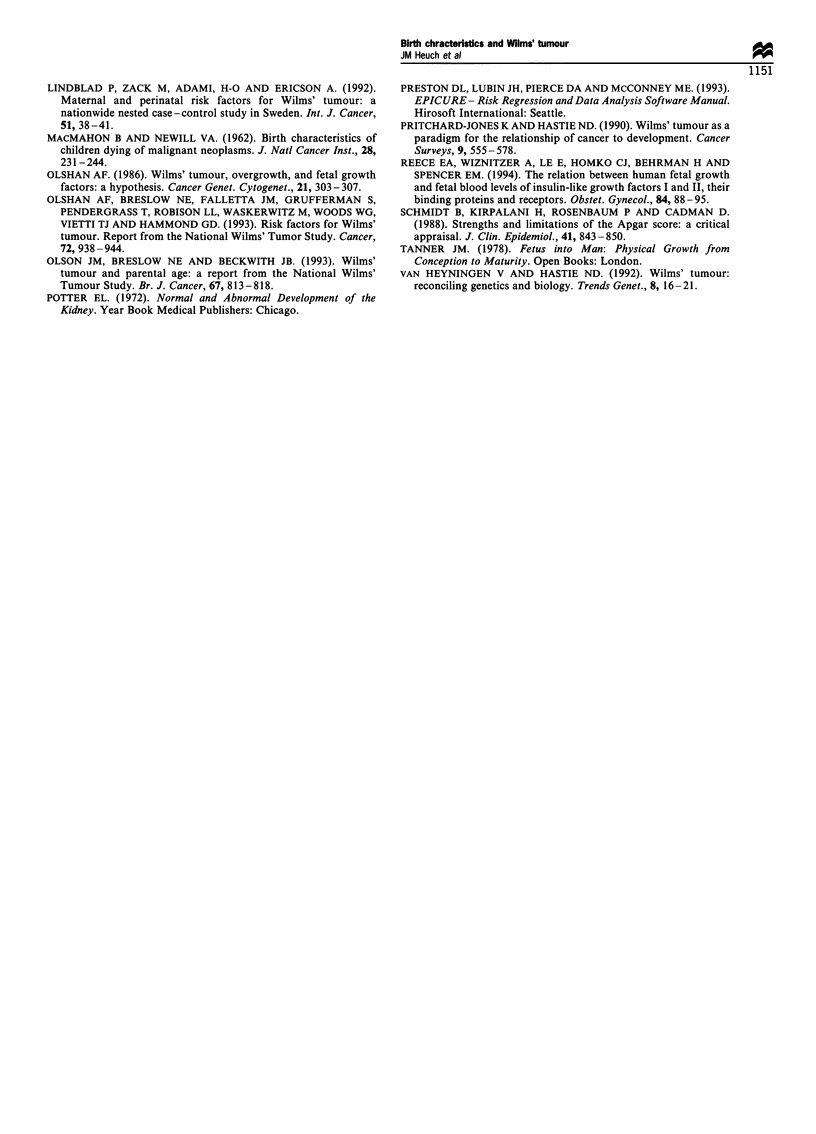# Birth characteristics and risk of Wilms' tumour: a nationwide prospective study in Norway.

**DOI:** 10.1038/bjc.1996.505

**Published:** 1996-10

**Authors:** J. M. Heuch, I. Heuch, G. Kvåle

**Affiliations:** Section for Medical Informatics and Statistics, University of Bergen, Norway.

## Abstract

Relationships between incidence of Wilms' tumour and information recorded at birth were investigated in a prospective study of the 1,489,297 children born in Norway between 1967 and 1992. A total of 119 individuals were diagnosed with Wilms' tumour in the age interval 0-14 years. A high length at birth was significantly associated with a high risk (incidence rate ratio 1.8 for length > or = 53 cm vs < or = 49 cm, 95% CI 1.0-3.2). A low Apgar score at 1 min was also associated with an increased risk (incidence rate ratio 2.2 for Apgar score < or = 8 vs a score > or = 9, 95% CI 1.2-3.9). For all variables for which an association was indicated, the association seemed to be restricted mainly to children aged less than 2 years. This suggests that Wilms' tumour diagnosed early in life may differ aetiologically from that of cases diagnosed later.


British Journal of Cancer (1996) 74, 1148-1151
© 1996 Stockton Press All rights reserved 0007-0920/96 $12.00

### Birth characteristics and risk of Wilms' tumour: a nationwide prospective study in Norway

JM Heuch[1], I Heuch[2] and G Kvåle[3]

[1]*Section for Medical Informatics and Statistics, University of Bergen, Armauer Hansen's Building, N-5021 Bergen, Norway;* [2]*Department of Mathematics, University of Bergen, N-5007 Bergen, Norway;* [3]*Centre for International Health, University of Bergen, Armauer Hansen's Building, N-5021 Bergen, Norway.*

**Summary** Relationships between incidence of Wilms' tumour and information recorded at birth were investigated in a prospective study of the 1 489 297 children born in Norway between 1967 and 1992. A total of 119 individuals were diagnosed with Wilms' tumour in the age interval 0-14 years. A high length at birth was significantly associated with a high risk (incidence rate ratio 1.8 for length ≥53 cm *vs* ≤49 cm, 95% CI 1.0-3.2). A low Apgar score at 1 min was also associated with an increased risk (incidence rate ratio 2.2 for Apgar score ≤8 *vs* a score ≥9, 95% CI 1.2-3.9). For all variables for which an association was indicated, the association seemed to be restricted mainly to children aged less than 2 years. This suggests that Wilms' tumour diagnosed early in life may differ aetiologically from that of cases diagnosed later.

**Keywords:** kidney neoplasm; childhood cancer; length of newborn; Apgar score; age interaction

Wilms' tumour is an embryonic tumour of the kidney and the majority of cases are diagnosed in early childhood (Breslow *et al.*, 1988). A recent study of the histology of Wilms' tumour (Beckwith *et al.*, 1990) showed that the tumour is generally composed of metanephric blastemal cells at various stages of differentiation. For a normal kidney to develop, differentiation of such cells must occur without interruption between weeks 5 and 35 of gestation (Potter, 1972). Thus, the persistence of undifferentiated cells suggests that a disturbance occurred in the development of the kidney during this period in patients with Wilms' tumour.

Certain paternal occupations have been implicated in the Getiology of Wilms' tumour but the evidence is not conclusive (Breslow *et al.*, 1993). Relations have also been explored with environmental and gestational variables and birth characteristics to ascertain factors which might predispose the fetus to the development of Wilms' tumour (Bunin *et al.*, 1987; Lindblad *et al.*, 1992; Olshan *et al.*, 1993). No final conclusions, however, can yet be drawn.

This paper investigates relations between birth characteristics and incidence of Wilms' tumour in a population-based prospective study. Particular attention is paid to the strength of the potential associations in the separate age groups less than 2 and 2-14 years.

### Materials and methods

The study population includes all 1 489 297 children born in Norway between 1967 and 1992. Registration with the Medical Birth Registry of Norway has been a legal requirement since 1967. A standard form, recording information about the birth and pregnancy, is completed by the midwife at the time of birth. The information includes birth date, sex, birth weight, length and vital status of the infant, Apgar score at 1 and at 5 min and any obvious birth defects or congenital malformations. The birth order of the infant is reported by the mother. Information is also recorded on the duration of the pregnancy, health problems of the mother before and during pregnancy, procedures used during delivery, use of analgesics etc., and demographic characteristics of the parents.

On the basis of the unique personal identification numbers used in Norway, the information from the Medical Birth Registry was linked to files from the Cancer Registry of Norway. Since 1953, all cases of cancer diagnosed in Norway must be reported to the Cancer Registry. For each child included in this study, the follow-up period extended from the date of birth until the child attained either the age of 15 years or the final date of follow-up, 31 December 1993; or was diagnosed with Wilms' tumour, or died of any cause. The follow-up comprised a total of 16 607 130 person-years, with a mean follow-up time per child of 11.2 years. The cases considered in this paper represent the 119 live-born children who were diagnosed during the follow-up with a histologically confirmed Wilms' tumour (ICD seventh revision site code 180.0). None of the cases had been diagnosed with the rare birth defects, aniridia, Beckwith-Wiedemann syndrome or hemihypertrophy, which can be associated with Wilms' tumour (Beckwith *et al.*, 1990).

Log-linear Poisson regression analysis, taking into account the period of follow-up for each child in separate age categories (Breslow and Day, 1987), was used to investigate associations between variables recorded in the Medical Birth Registry and the incidence of Wilms' tumour. The computations were performed using the program package Epicure (Preston *et al.*, 1993). Adjustment was made for sex and age, with 1-year categories until the age of 6 years and a combined category for the low-risk age group 6-14 years. In view of the heterogeneity in the pathology of Wilms' tumour, with different subtypes tending to predominate among younger or older patients (Beckwith *et al.*, 1990; Breslow *et al.*, 1993), the age intervals 0-1 years and 2-14 years were also analysed separately. Likelihood ratio tests for differences in risk were based on the regression analyses. Certain analyses included only a subset of the complete data set as the potential risk factor involved had not been recorded for all children in the Medical Birth Registry. The relevant number of cases in each risk category is given in the tables.

### Results

The risk of Wilms' tumour was strongly associated with the age of the child (Table I). A peak in risk was seen in the bimodal age distribution during the second year, with a much lower peak in the fifth year. The risk declined sharply after the sixth year.

A total of 63 boys and 56 girls were diagnosed with Wilms' tumour. The median age at diagnosis was 26 months for boys and 35 months for girls. The rate ratio for boys *vs*

Correspondence: JM Heuch
Received 2 February 1996; revised 9 May 1996; accepted 9 May 1996

girls differed somewhat between age groups (Table I). Risk estimates were higher for boys than for girls during the first two years, with a reversed relationship at higher ages. A test for interaction between sex and age (considering the broad categories 0–23 months and ⩾24 months) was marginally significant ($P=0.086$).

Higher risk estimates were found with increasing gestational age (Table II), although no statistically significant relation could be established. Birth weight showed no clear overall association with risk of Wilms' tumour, despite an indication that infants weighing more than 4000 g at birth carried a slightly increased risk. In contrast, children who were 52 cm or longer at birth had a significantly elevated risk (Table II). Risk estimates for length were essentially unchanged by adjustment for birth weight, while associations suggested for birth weight were weakened by adjustment for length (results not shown). Infants with an Apgar score at 1 min of 8 or less carried a 2-fold risk of Wilms' tumour. Similar, but not as marked, results were obtained for Apgar score at 5 min [with an incidence rate ratio (IRR) of 1.49 for a score of ⩽9 *vs*⩾10, 95% CI 0.81–2.72]. The relation with Apgar score was unaffected by further adjustment for body weight or length. Neither mother's age nor father's age were associated with risk of Wilms' tumour (Table II). No association was found with birth order (IRR=1.01 for birth order ⩾2 *vs* ⩽1, 95% CI 0.66–1.54) or time since previous birth for the mother (IRR=0.86 for >24 months *vs* ⩽24 months, 95% CI 0.45–1.66).

The estimated rate ratio for Wilms' tumour for children with complications during birth reported for placenta, amniotic fluid or umbilical cord was 1.03 (95% CI 0.67–1.58), with all children without any reports of such complications as the reference group. The corresponding rate ratio for complications at birth involving the fetus was 0.78 (95% CI 0.38–1.61), for anaesthetics used at birth 0.71 (95% CI 0.40–1.27), for induced birth 0.94 (95% CI 0.56–1.60), and for complications at birth involving the mother 0.91 (95% CI 0.51–1.62). Infants, whose mothers reported problems with their health during pregnancy, had a rate ratio of 0.89 (95% CI 0.50–1.58).

Table III gives results of analyses of potential linear relations between risk of Wilms' tumour and the variables gestational age, birth weight, length and Apgar score at 1 min. The results are given separately for the age groups <24 and⩾ 24 months, as well as for the entire age range. For each variable considered, a stronger linear association was found for children aged less than 24 months. For both length and Apgar score at 1 min, the associations were statistically significant among the very young children but not in the higher age group. For birth weight, an increased risk was found among children less than 24 months in the particular category corresponding to weights above 4000 g (IRR=2.21, 95% CI 1.02–4.81, compared with weights in the interval 3001–3500 g, represented by 16 and 13 cases respectively).

**Table I** Risk of Wilms' tumour by age, prospective study, Norway 1967–1993

| | *No. of cases* | *Incidence rate*[a] *Total* | *Males* | *Females* | *Incidence rate ratio males* vs *females* |
|---|---|---|---|---|---|
| Age (months) | | | | | |
| 0–11 | 22 | 14.9 | 18.5 | 11.1 | 1.66 |
| 12–23 | 26 | 18.0 | 21.6 | 14.2 | 1.52 |
| 24–35 | 23 | 16.6 | 16.9 | 16.3 | 1.04 |
| 36–47 | 10 | 7.6 | 4.4 | 10.9 | 0.41 |
| 48–59 | 15 | 11.9 | 10.8 | 13.0 | 0.83 |
| 60–71 | 11 | 9.1 | 8.1 | 10.2 | 0.79 |
| 72–179 | 12 | 1.4 | 1.4 | 1.4 | 0.95 |

[a]Per $10^6$ person–years

### Discussion

In this population-based prospective study involving perinatal risk factors, the incidence of Wilms' tumour was found to be associated with a high length at birth and a low Apgar score

**Table II** Risk of Wilms' tumour by birth characteristics, prospective study, Norway 1967–1993[a]

| | *No. of cases* | *Incidence rate ratio (with 95% CI)* | P-*value for trend* |
|---|---|---|---|
| Gestational age (weeks) | | | 0.20 |
| ⩽37 | 7 | 0.66 (0.29–1.50) | |
| 38–39 | 27 | 0.81 (0.49–1.35) | |
| 40 | 33 | 1.00[b] | |
| 41–42 | 34 | 0.90 (0.56–1.45) | |
| ⩾43 | 8 | 1.47 (0.68–3.18) | |
| Birth weight (g) | | | 0.22 |
| ⩽3000 | 12 | 0.63 (0.33–1.20) | |
| 3001–3500 | 40 | 1.00[b] | |
| 3501–4000 | 33 | 0.78 (0.49–1.25) | |
| >4000 | 24 | 1.19 (0.72–1.98) | |
| Length (cm) | | | 0.025 |
| ⩽49 | 26 | 1.00[b] | |
| 50 | 24 | 1.39 (0.80–2.43) | |
| 51 | 22 | 1.43 (0.81–2.53) | |
| 52 | 22 | 1.81 (1.02–3.20) | |
| ⩾53 | 23 | 1.79 (1.01–3.16) | |
| Apgar score at 1 min | | | 0.022 |
| ⩽7 | 7 | 2.15 (0.95–4.87) | |
| 8 | 11 | 2.20 (1.11–4.35) | |
| 9, 10 | 33 | 1.00[b] | |
| Mother's age at birth (years) | | | 0.97 |
| ⩽20 | 14 | 1.00[b] | |
| 21–25 | 43 | 1.17 (0.64–2.13) | |
| 26–30 | 40 | 1.23 (0.67–2.27) | |
| ⩾31 | 22 | 1.03 (0.52–2.01) | |
| Father's age at birth (years) | | | 0.29 |
| ⩽25 | 37 | 1.00[b] | |
| 26–30 | 37 | 0.78 (0.49–1.23) | |
| 31–35 | 27 | 0.91 (0.55–1.50) | |
| ⩾36 | 11 | 0.65 (0.35–1.23) | |

[a]Results adjusted for age and sex. [b]Reference category.

**Table III** Linear relations with incidence of Wilms' tumour, by age, prospective study, Norway 1967–1993[a]

| *Linear variable* | *Age group (years)* | *No. of cases* | *Incidence rate ratio (with 95% CI) based on linear relation*[b] | P-*value for linear interaction with age*[c] |
|---|---|---|---|---|
| Gestational age | | | | 0.97 |
| | 0–1 | 44 | 1.17 (0.88–1.55) | |
| | 2–14 | 65 | 1.09 (0.87–1.38) | |
| | Total | 109 | 1.12 (0.94–1.34) | |
| Birth weight | | | | 0.09 |
| | 0–1 | 44 | 1.30 (0.95–1.80) | |
| | 2–14 | 65 | 1.03 (0.80–1.34) | |
| | Total | 109 | 1.13 (0.93–1.38) | |
| Length | | | | 0.027 |
| | 0–1 | 47 | 1.28 (1.05–1.57) | |
| | 2–14 | 70 | 1.07 (0.91–1.27) | |
| | Total | 117 | 1.16 (1.02–1.31) | |
| Apgar score at 1 min | | | | 0.21 |
| | 0–1 | 23 | 0.53 (0.33–0.88) | |
| | 2–14 | 58 | 0.75 (0.45–1.28) | |
| | Total | 51 | 0.64 (0.44–0.91) | |

[a]Results adjusted for age and sex. [b]IRR for comparing successive levels of linear variable, using categories shown in Table II. [c]*P*-value for linear change in association, over all categories of age shown in Table I.

at 1 min. The associations were mainly restricted to children aged less than 2 years. The study design eliminates selection bias but the possibility of information bias should still be considered for certain variables. The data from the Medical Birth Registry are standardised but information may be incomplete for variables such as structure of placenta or description of the amniotic fluid. For this reason, only broad groups were considered in the evaluation of risk for such factors. The limited number of cases makes it difficult to assess associations in subgroups of the data set. To increase the statistical power, such analyses were mainly based on potential linear relations among grouped observations.

The general age distribution of the cases is in agreement with observations from other Western countries (Breslow *et al.*, 1993). The higher age at diagnosis in females than in males is consistent with registry data from the United States (Breslow *et al.*, 1993), although the median age for each sex seems to be lower in Norway. In contrast to the results of Olson *et al.* (1993), based on a much larger data set, we found no relation with parental age. However, because of the age range (17–42 years for mothers and 18–46 years for fathers of cases), we were not able to study associations with the very high parental ages for which Olson *et al.* (1993) found an increased risk.

No significant association was observed with birth weight in our analyses including all children aged less than 15 years. Similar results were found by MacMahon and Newill (1962), Bunin *et al.* (1987), Lindblad *et al.* (1992) and Olshan *et al.* (1993). Our risk estimates indicate, however, that children under the age of 2 with a birth weight exceeding 4000 g may carry an increased risk. This is in agreement with the results of Daling *et al.* (1984), but not those of Bunin *et al.* (1987) and Olshan *et al.* (1993).

In contrast to the results of Lindblad *et al.* (1992), we found a statistically significant trend with an increasing risk of Wilms' tumour over the whole range of length measured at birth. The association was particularly strong among children with a diagnosis before the age of 2 years but largely disappeared in the older age groups. Olshan (1986) suggested that the high birth weight seen in some children with Wilms' tumour might be related to an abnormal interaction of the IGF-II (insulin-like growth factor II) gene and possibly the insulin gene with the Wilms' tumour gene, WT1. Van Heyningen and Hastie (1992) hypothesised that the maternal allele loss at the second chromosome region 11p15, implicated in the development of Wilms' tumour, might be associated with increased growth-promoting activity caused by two parternally derived copies of IGF-II. These hypotheses may also be relevant to the association seen with high length. The maximum growth acceleration in terms of fetal skeletal growth occurs around 16–18 weeks of gestation (Tanner, 1978) and IGF-I and IGF-II are thought to be important growth factors at this stage (Reece *et al.*, 1994).

In this study, no association was found with the administration of anaesthetics during delivery. Thus, we are unable to support the finding that administration of penthrane during delivery is related to an increased risk of Wilms' tumour (Lindblad *et al.*, 1992). However, under-reporting of use of anaesthetics to the Medical Birth Registry may have influenced our risk estimate.

A high risk of Wilms' tumour was observed among children with a lowered Apgar score at 1 min, with a similar but weaker relation for Apgar score at 5 min. The Apgar score was available only for a subset of the children considered. The risk of Wilms' tumour among children with a missing Apgar score at 1 min was intermediate (IRR = 1.56 compared with children with an Apgar score of 9 or 10). Thus, it is difficult to see how any substantial bias in the association with subsequent tumour diagnosis could be introduced by under-reporting. The Apgar score is based on evaluation of the newborn's heart rate, respiratory effort, reflex ability, muscle tone and colour (Schmidt *et al.*, 1988) and a low score is in general an indicator of a stressed birth. Since the relation with Apgar score was unaffected by adjustment for birth weight and for length, it could reflect an underlying developmental defect associated with Wilms' tumour, especially in the cases diagnosed during the first 2 years of life.

More generally, however, associations in our data set seemed to be restricted to Wilms' tumour diagnosed early in life. There are several indications that the aetiology of Wilms' tumour may differ between early and late cases, with a change occurring at about 2–3 years of age (Beckwith *et al.*, 1990; Breslow *et al.*, 1993). This may reflect disturbances in the nephrogenic process occurring in different phases of development (Pritchard-Jones and Hastie, 1990). With regard to precursor lesions found in connection with Wilms' tumour, cases with an early diagnosis tend in general to be associated with intralobar nephrogenic rests, whereas a late diagnosis is often associated with perilobar rests (Breslow *et al.*, 1993). The two types also tend to be associated with different kinds of congenital anomalies (Beckwith *et al.*, 1990). The relations with perinatal risk factors seen in our data set may represent the early type of Wilms' tumour, associated with disturbances before week 14 during pregnancy (Pritchard-Jones and Hastie, 1990), the end of the first phase in the development of the kidney (Potter, 1972). In contrast, the later type may have an aetiology which is not reflected in associations with variables recorded at birth.

Assuming that an essential disturbance in the development of the kidney took place before 14 weeks of gestation, variables registered at birth are expected to shed information on an event which happened at least 26 weeks earlier. In order to collect more informative data on potential risk factors during the pregnancy, we suggest that the relevant earlier periods should be monitored with greater detail. For the tumours diagnosed in older children, possibly other environmental factors play a stimulating role in the transformation from the susceptible state to the malignant.

**Acknowledgements**
This work was funded by the Norwegian Cancer Society. The authors thank the Cancer Registry of Norway and the Medical Birth Registry of Norway for giving them access to the data. The authors are grateful to Dr Lars A Akslen for his constructive comments.

### References

BECKWITH JB, KIVIAT NB AND BONADIO JF. (1990). Nephrogenic rests, nephroblastomatosis, and the pathogenesis of Wilms' tumor. *Pediatr. Pathol.*, **10**, 1–36.

BRESLOW NE AND DAY NE. (1987). *Statistical Methods in Cancer Research*. Vol. 2, *The Design and Analysis of Cohort Studies*, IARC Scientific Publications No. 82. IARC: Lyon.

BRESLOW NE, BECKWITH JB, CIOL M AND SHARPLES K. (1988). Age distribution of Wilms' tumour: report from the National Wilms' tumour study. *Cancer Res.*, **48**, 1653–1657.

BRESLOW NE, OLSHAN AF, BECKWITH JB AND GREEN DM. (1993). Epidemiology of Wilms' tumor. *Med. Pediatr. Oncol.*, **21**, 172–181.

BUNIN GR, KRAMER S, MARRERO O AND MEADOWS AT. (1987). Gestational risk factors for Wilms' tumour: results of a case–control study. *Cancer Res.*, **47**, 2972–2977.

DALING JR, STARZYK P, OLSHAN AF AND WEISS NS. (1984). Birth weight and the incidence of childhood cancer. *J. Natl Cancer Inst.*, **72**, 1039–1041.

LINDBLAD P, ZACK M, ADAMI, H-O AND ERICSON A. (1992). Maternal and perinatal risk factors for Wilms' tumour: a nationwide nested case–control study in Sweden. *Int. J. Cancer*, **51,** 38–41.

MACMAHON B AND NEWILL VA. (1962). Birth characteristics of children dying of malignant neoplasms. *J. Natl Cancer Inst.*, **28,** 231–244.

OLSHAN AF. (1986). Wilms' tumour, overgrowth, and fetal growth factors: a hypothesis. *Cancer Genet. Cytogenet.*, **21,** 303–307.

OLSHAN AF, BRESLOW NE, FALLETTA JM, GRUFFERMAN S, PENDERGRASS T, ROBISON LL, WASKERWITZ M, WOODS WG, VIETTI TJ AND HAMMOND GD. (1993). Risk factors for Wilms' tumour. Report from the National Wilms' Tumor Study. *Cancer*, **72,** 938–944.

OLSON JM, BRESLOW NE AND BECKWITH JB. (1993). Wilms' tumour and parental age: a report from the National Wilms' Tumour Study. *Br. J. Cancer*, **67,** 813–818.

POTTER EL. (1972). *Normal and Abnormal Development of the Kidney*. Year Book Medical Publishers: Chicago.

PRESTON DL, LUBIN JH, PIERCE DA AND MCCONNEY ME. (1993). *EPICURE – Risk Regression and Data Analysis Software Manual.* Hirosoft International: Seattle.

PRITCHARD-JONES K AND HASTIE ND. (1990). Wilms' tumour as a paradigm for the relationship of cancer to development. *Cancer Surveys*, **9,** 555–578.

REECE EA, WIZNITZER A, LE E, HOMKO CJ, BEHRMAN H AND SPENCER EM. (1994). The relation between human fetal growth and fetal blood levels of insulin-like growth factors I and II, their binding proteins and receptors. *Obstet. Gynecol.*, **84,** 88–95.

SCHMIDT B, KIRPALANI H, ROSENBAUM P AND CADMAN D. (1988). Strengths and limitations of the Apgar score: a critical appraisal. *J. Clin. Epidemiol.*, **41,** 843–850.

TANNER JM. (1978). *Fetus into Man: Physical Growth from Conception to Maturity*. Open Books: London.

VAN HEYNINGEN V AND HASTIE ND. (1992). Wilms' tumour: reconciling genetics and biology. *Trends Genet.*, **8,** 16–21.